# Supplementary material for: Continuous Fiber-Reinforced Aramid/PETG 3D-Printed Composites with High Fiber Loading through Fused Filament Fabrication
Source: Polymers (Basel). 2022 Jan 12;14(2):298. doi: 10.3390/polym14020298 (PMC8778918; doi:10.3390/polym14020298)
Supplement: Supplementary file 1 [file polymers-14-00298-s001.zip › polymers-1535167-supplementary.pdf]

Supporting Information

Continuous fiber reinforced aramid/PETG 3D printed composites with high fiber loadings through low-cost Fused Deposition Modelling.

Section S1: Mechanical properties of aramid fibers before and after printing

To determine whether the printing process had a deteriorating effect on the mechanical properties of the reinforcing aramid fibers, single fiber tensile tests were performed on samples before printing ("virgin" fibers) and after "dry" printing. During "dry" printing, an aramid fiber bundles was pulled through a printer heated to the same temperature as during printing of composite samples (230°C), at the same speed as during printing (150 mm/min). To mimic the effect of rubbing against the brass printing nozzle, fibers were pulled at an angle of 90°. Individual fibers were then tested to determine strength and modulus.

Figure S1 shows a comparison of representative stress/strain curves of both aramid fiber types. In Figure S2, the tensile modulus and ultimate tensile strength (UTS) derived from these tests is shown. While there seems to be a slight decrease in mechanical properties, there is no statistical difference in both modulus and UTS.

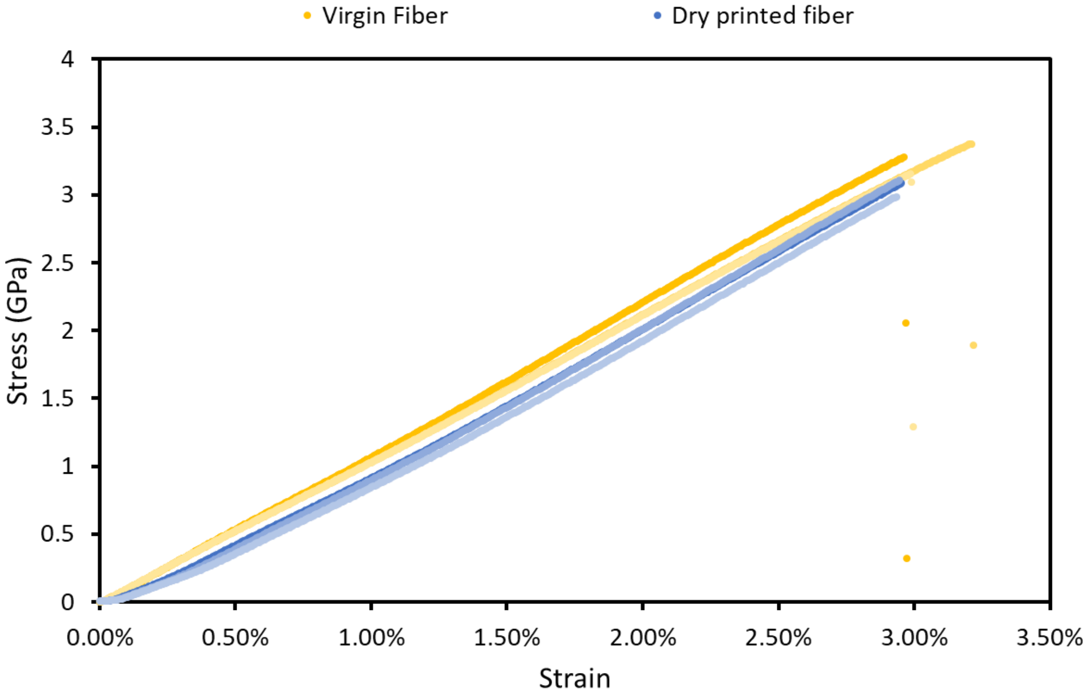

Figure S1 Comparison of representative stress/strain curves of 'virgin' aramid fibers and aramid fibers after 'dry printing'. There is no clear difference in mechanical behavior.

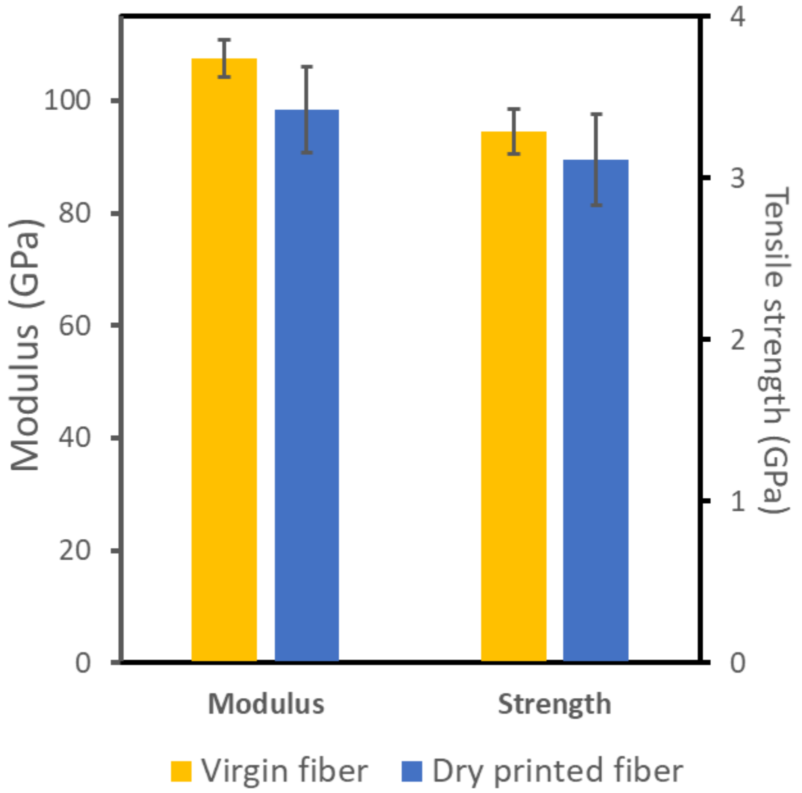

Figure S2 Comparison of modulus and tensile strength of “virgin” and “dry printed” aramid fibers. There is no statistically relevant difference between the results before and after printing.

Section S2: Effect of fiber addition on the thermal properties of the matrix polymer

Differential scanning calorimetry (DSC) was used to determine if the addition of fibers added a significant degree of degradation to the matrix polymer during printing. The glass transition temperature (Tg) of printed composite samples containing 0 vol%, 20 vol% and 40 vol% aramid fibers in PETG matrix was determined. Figure S3 shows the heat flow (collated) of these samples during the first heating cycle. Tg was determined using TRIOS software by TA instruments. While there is a slight difference in Tg between non-reinforced (80°C) and reinforced (77°C for both 20 vol% and 40 vol% samples), this is not enough to indicate significant degradation.

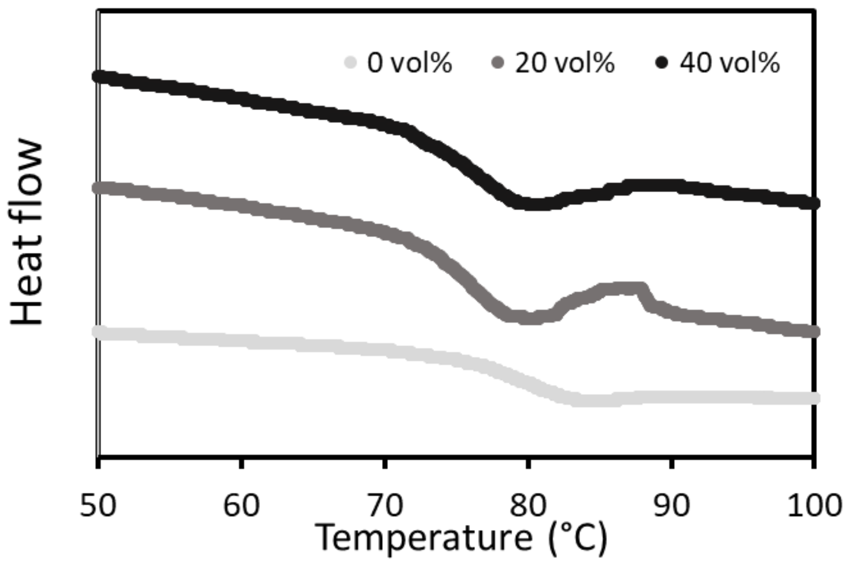

Figure S3 Collation of first heating cycles of 3D printed composites with different amount of aramid fibers. The Tg of the matrix polymers is slightly lower (76.5°C vs. 79.5°C), but this is not indicative of polymer degradation.

Section S3: Effect of printing parameters on composite inner structure

Optical microscopy was performed to gain insight on the inner structure of composites printed with different printing parameters. The line spacing and layer height were varied in order to obtain a layered structure typical for traditional composites in contrast to the array-like structure typical for 3D printing. This also had an effect on the presence of voids in between the printed lines.

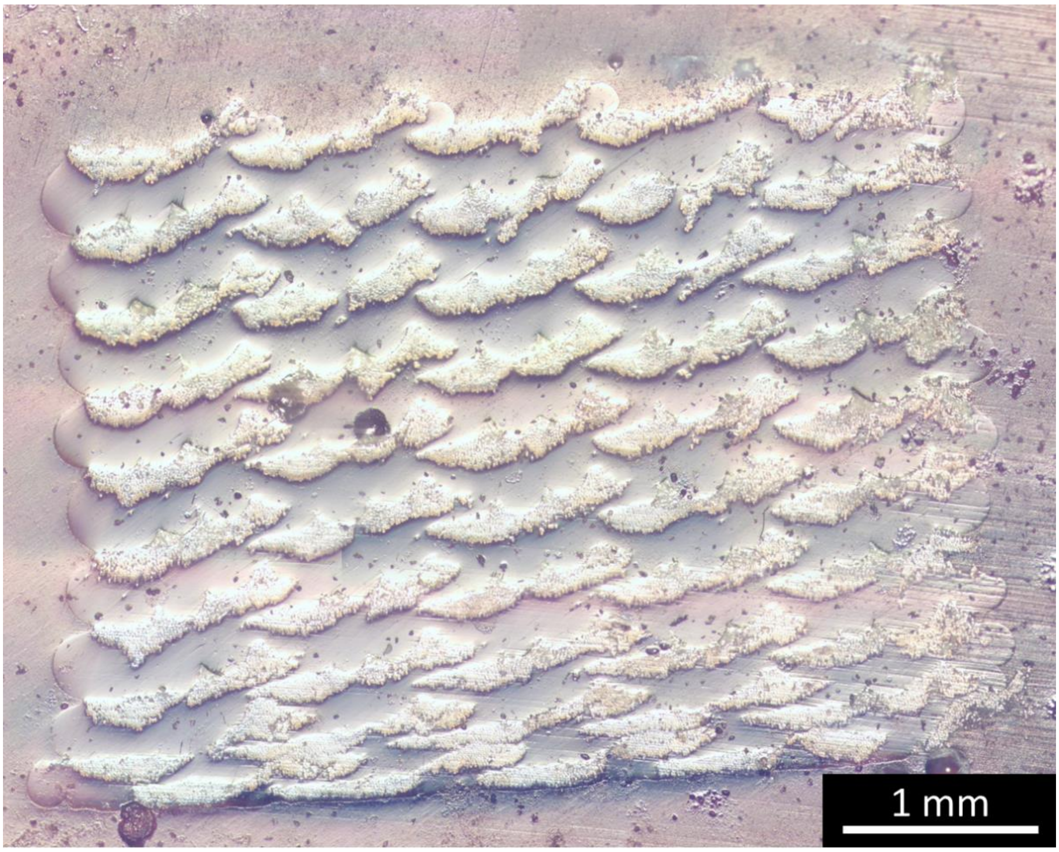

Figure S4 Micrograph of a printed composite with configuration A: 0.4 mm line spacing, 0.45 mm layer height. The overall fiber loading is 22 vol%.

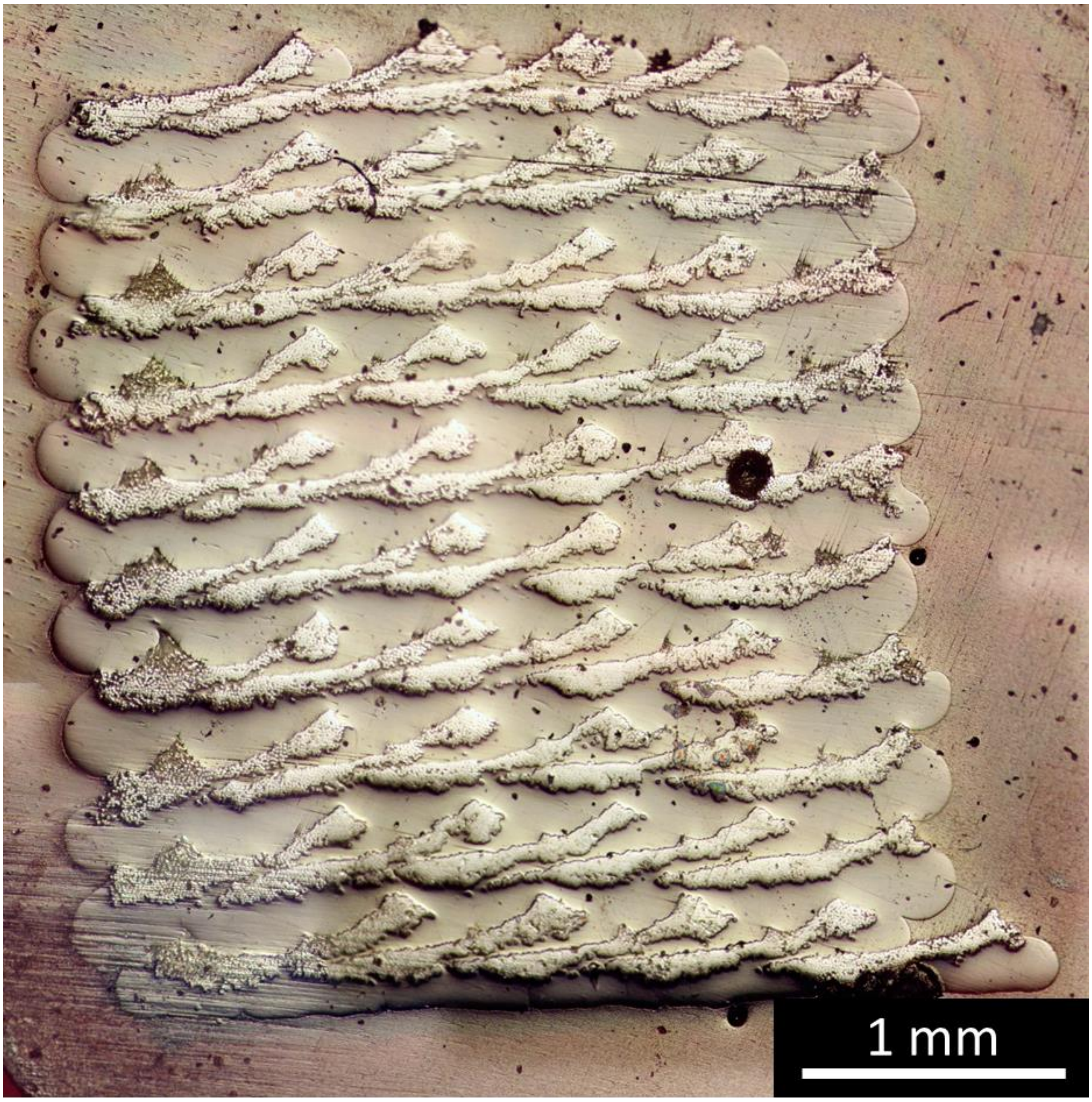

Figure S5 Micrograph of a printed composite with configuration B: 0.3 mm line spacing, 0.4 mm layer height. The overall fiber loading is 29 vol%.

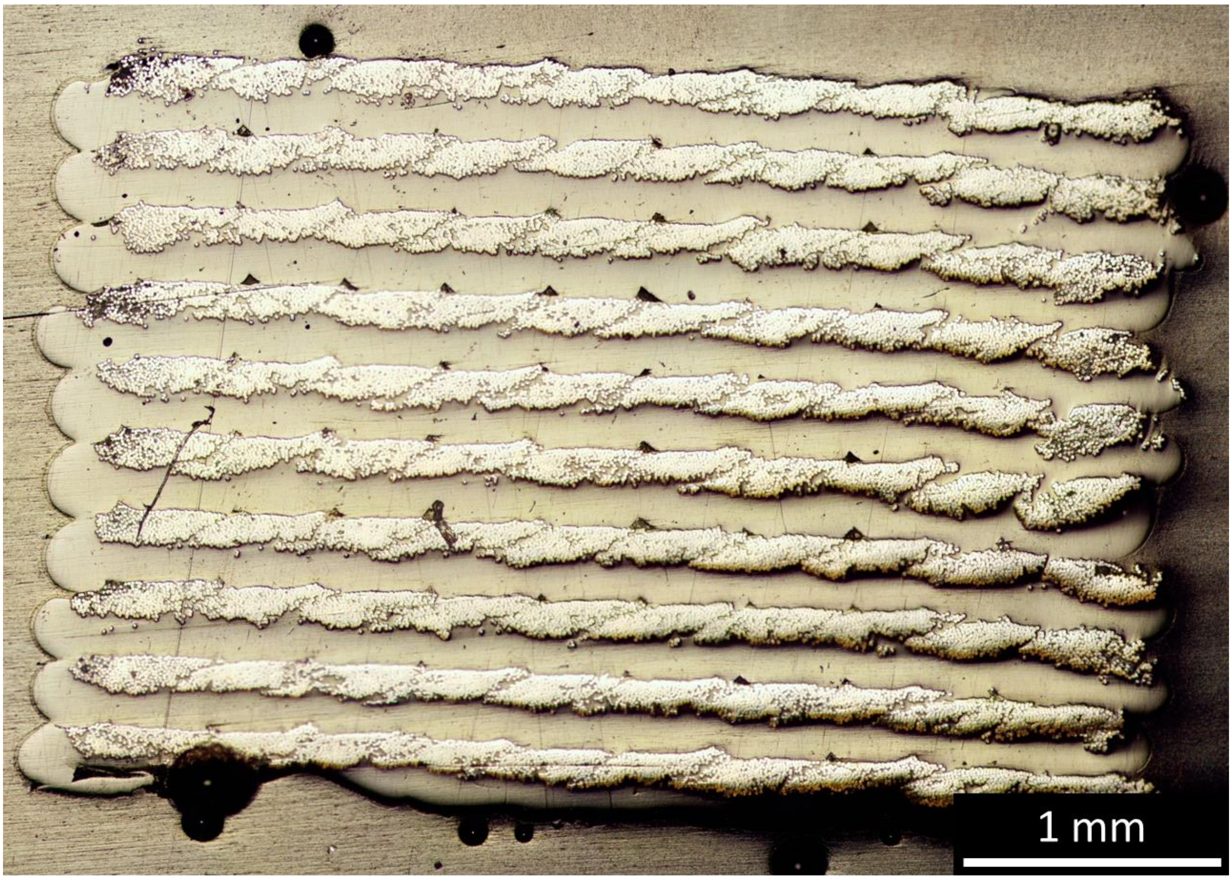

Figure S6 Micrograph of a printed composite with configuration C: 0.4 mm line spacing, 0.3 mm layer height. The overall fiber loading is 31 vol%.

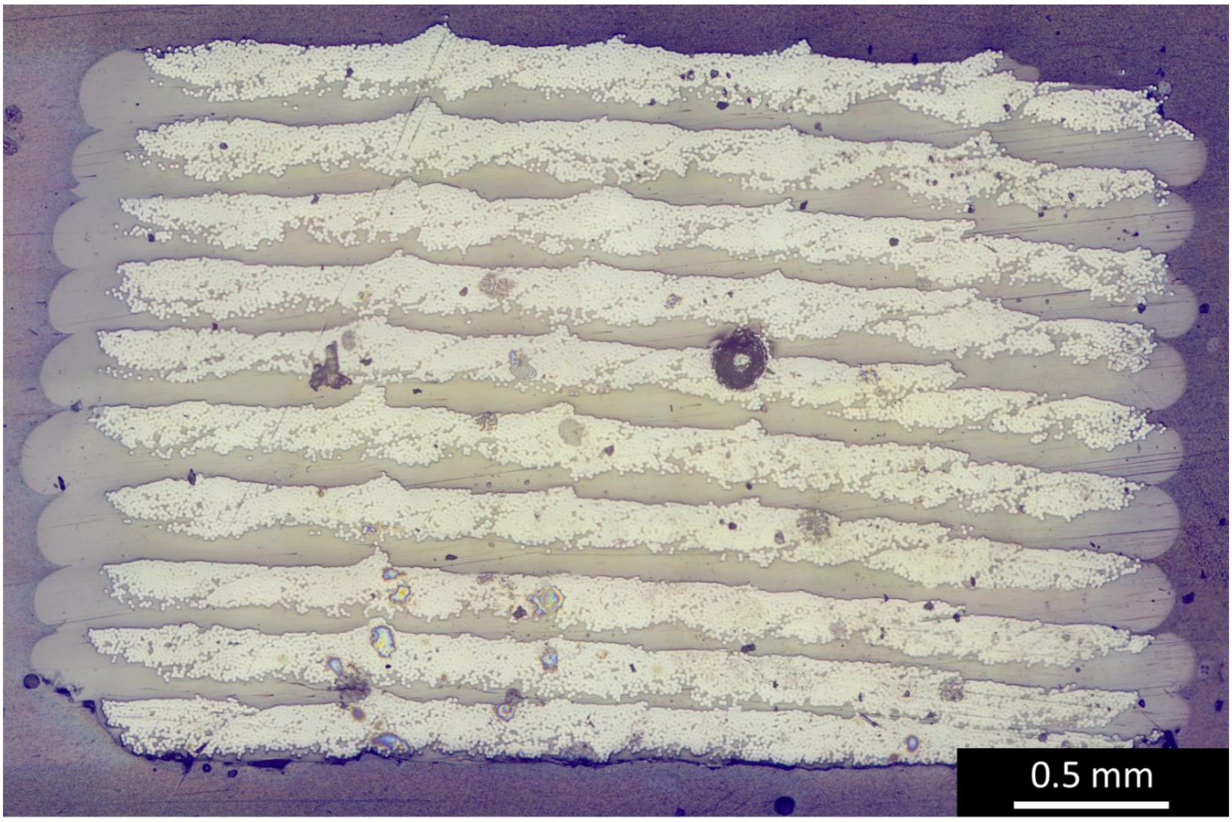

Figure S7 Micrograph of a printed composite with configuration D: 0.3 mm line spacing, 0.25 mm layer height. The overall fiber loading is 45 vol%.
